# Efficacy and Safety of Cannabidiol Plus Standard Care vs Standard Care Alone for the Treatment of Emotional Exhaustion and Burnout Among Frontline Health Care Workers During the COVID-19 Pandemic: A Randomized Clinical Trial

**DOI:** 10.1001/jamanetworkopen.2021.20603

**Published:** 2021-08-13

**Authors:** José Alexandre S. Crippa, Antonio W. Zuardi, Francisco S. Guimarães, Alline Cristina Campos, Flávia de Lima Osório, Sonia Regina Loureiro, Rafael G. dos Santos, José Diogo S. Souza, Juliana Mayumi Ushirohira, Julia Cozar Pacheco, Rafael Rinaldi Ferreira, Karla Cristinne Mancini Costa, Davi Silveira Scomparin, Franciele Franco Scarante, Isabela Pires-Dos-Santos, Raphael Mechoulam, Flávio Kapczinski, Benedito A. L. Fonseca, Danillo L. A. Esposito, Karina Pereira-Lima, Srijan Sen, Maristela Haddad Andraus, Jaime E. C. Hallak

**Affiliations:** 1 Department of Neuroscience and Behavior, Ribeirão Preto Medical School, University of São Paulo, Ribeirão Preto, São Paulo, Brazil; 2 National Institute for Science and Technology–Translational Medicine, São Paulo, Brazil; 3 Department of Pharmacology, Ribeirão Preto Medical School, University of São Paulo, Ribeirão Preto, São Paulo, Brazil; 4 Institute for Drug Research, School of Pharmacy, Hebrew University of Jerusalem, Jerusalem, Israel; 5 Department of Psychiatry and Behavioural Neurosciences, McMaster University, Hamilton, Ontario, Canada; 6 Department of Psychiatry, Faculty of Medicine, Graduate Program in Psychiatry and Behavioral Sciences, Universidade Federal do Rio Grande do Sul, Porto Alegre, Brazil; 7 Department of Internal Medicine, Ribeirão Preto Medical School, University of São Paulo, Ribeirão Preto, São Paulo, Brazil; 8 Department of Psychiatry, University of Michigan Medical School, Ann Arbor; 9 Department of Psychiatry, Federal University of São Paulo, São Paulo, Brazil; 10 Chromatox Laboratory, São Paulo, Brazil

## Abstract

**Importance:**

Frontline health care professionals who work with patients with COVID-19 have an increased incidence of burnout symptoms. Cannabidiol (CBD) has anxiolytic and antidepressant properties and may be capable of reducing emotional exhaustion and burnout symptoms.

**Objective:**

To investigate the safety and efficacy of CBD therapy for the reduction of emotional exhaustion and burnout symptoms among frontline health care professionals working with patients with COVID-19.

**Design, Setting, and Participants:**

This prospective open-label single-site randomized clinical trial used a 1:1 block randomization design to examine emotional exhaustion and burnout symptoms among frontline health care professionals (physicians, nurses, and physical therapists) working with patients with COVID-19 at the Ribeirão Preto Medical School University Hospital in São Paulo, Brazil. Participants were enrolled between June 12 and November 12, 2020. A total of 214 health care professionals were recruited and assessed for eligibility, and 120 participants were randomized in a 1:1 ratio by a researcher who was not directly involved with data collection.

**Interventions:**

Cannabidiol, 300 mg (150 mg twice per day), plus standard care or standard care alone for 28 days.

**Main Outcomes and Measures:**

The primary outcome was emotional exhaustion and burnout symptoms, which were assessed for 28 days using the emotional exhaustion subscale of the Brazilian version of the Maslach Burnout Inventory–Human Services Survey for Medical Personnel.

**Results:**

A total of 120 participants were randomized to receive either CBD, 300 mg, plus standard care (treatment arm; n = 61) or standard care alone (control arm; n = 59) for 28 days. Of those, 118 participants (59 participants in each arm; 79 women [66.9%]; mean age, 33.6 years [95% CI, 32.3-34.9 years]) received the intervention and were included in the efficacy analysis. In the treatment arm, scores on the emotional exhaustion subscale of the Maslach Burnout Inventory significantly decreased at day 14 (mean difference, 4.14 points; 95% CI, 1.47-6.80 points; partial eta squared [η_p_^2^] = 0.08), day 21 (mean difference, 4.34 points; 95% CI, 0.94-7.73 points; η_p_^2^ = 0.05), and day 28 (mean difference, 4.01 points; 95% CI, 0.43-7.59 points; η_p_^2^ = 0.04). However, 5 participants, all of whom were in the treatment group, experienced serious adverse events: 4 cases of elevated liver enzymes (1 critical and 3 mild, with the mild elevations reported at the final 28-day assessment) and 1 case of severe pharmacodermia. In 2 of those cases (1 with critical elevation of liver enzymes and 1 with severe pharmacodermia), CBD therapy was discontinued, and the participants had a full recovery.

**Conclusions and Relevance:**

In this study, CBD therapy reduced symptoms of burnout and emotional exhaustion among health care professionals working with patients during the COVID-19 pandemic. However, it is necessary to balance the benefits of CBD therapy with potential undesired or adverse effects. Future double-blind placebo-controlled clinical trials are needed to confirm the present findings.

**Trial Registration:**

ClinicalTrials.gov Identifier: NCT04504877

## Key Points

**Question:**

Is cannabidiol (CBD) therapy capable of reducing emotional exhaustion and burnout symptoms among frontline health care professionals working with patients with COVID-19?

**Findings:**

In this randomized clinical trial of 120 frontline health care professionals, emotional exhaustion scores were reduced among participants receiving CBD plus standard care compared with those receiving standard care alone. Five participants who received CBD plus standard care experienced serious adverse events, with full recovery after discontinuation.

**Meaning:**

The study’s findings suggest that CBD may act as an effective agent for the reduction of emotional exhaustion and burnout symptoms among frontline health care professionals, although it is necessary to balance the benefits with potential undesired effects when making decisions regarding the use of CBD.

## Teaser

This randomized clinical trial assesses the safety and efficacy of cannabidiol plus standard care vs standard care alone for the treatment of emotional exhaustion and burnout symptoms among frontline health care professionals working with patients with COVID-19.

## Introduction

During the COVID-19 pandemic, most countries adopted lockdown and physical distancing measures as containment strategies. This situation led to severe social and economic consequences and affected mental health.^1,2,3,4^ Several surveys have reported pandemic-related increases in emotional distress, depression,^3,5^ anxiety,^3,5^ posttraumatic stress disorder (PTSD),^3,4,5,6^ and insomnia,^6^ particularly among frontline health care workers.^2^ The usual pharmacological treatments for these conditions (antidepressant, anxiolytic, and hypnotic medications) often require several weeks to be effective and can produce substantial adverse effects (AEs). Safer and more effective drugs to treat the symptoms of emotional exhaustion and burnout are needed, especially during the current COVID-19 pandemic.

Cannabidiol (CBD) is a nonpsychotomimetic phytocannabinoid with a favorable safety and tolerability profile. The drug has been reported to have anxiolytic effects in healthy volunteers^7,8,9,10^ and patients with social anxiety disorder^11^ at doses ranging from a single administration of 300 to 600 mg^12,13,14,15,16^ to daily administration of 300 mg for 4 weeks.^17^ Cannabidiol also been found to have antidepressant^12,13,14^ and antiinflammatory^15,16,17,18,19,20,21^ effects in preclinical studies.

Considering the potential beneficial properties of CBD and its favorable safety profile, we assessed the efficacy and safety of daily administration of CBD therapy to decrease the symptoms of emotional exhaustion and burnout among frontline health care workers treating patients with COVID-19 in a Brazilian hospital. Our secondary outcomes were to evaluate anxiety, depression, and PTSD symptoms and to examine proinflammatory cytokine levels and general laboratory measurements.

## Methods

### Design

The Burnout and Distress Prevention With Cannabidiol in Front-line Health Care Workers Dealing With COVID-19 (BONSAI) study was a single-site 2-arm parallel-group randomized clinical trial designed to assess whether the efficacy and safety of oral CBD, 300 mg, plus standard care administered daily for 28 days was superior to standard care alone for the prevention or reduction of emotional exhaustion and burnout symptoms among health care professionals working with patients with COVID-19. Evaluators were blinded, whereas participants and investigators were not. After the initial 28-day period, all participants were offered continuation of treatment with CBD, 300 mg, for an additional 28 days. The present article presents only the data collected before this open-label extension period.

The initial project had a placebo-controlled design. However, because it would have been challenging to conduct a clinical trial of a new pharmacological intervention among professionals who were working directly with patients with severe cases of COVID-19 in potentially high-risk situations, the local ethics committee recommended alteration to an open-blind design with blind rater assessments. The trial protocol was submitted and approved by the research ethics committee of the Ribeirão Preto Medical School University Hospital and the National Council on Research Ethics (Supplement 1). Before enrollment and randomization, written informed consent was obtained from all participants. An independent data safety monitoring committee periodically reviewed the safety of the entire clinical program, including test abnormalities. The study adhered to the Declaration of Helsinki,^22^ the *Guideline for Good Clinical Practice*,^23^ and local regulatory requirements, and it followed the Consolidated Standards of Reporting Trials (CONSORT) reporting guideline for randomized clinical trials.

Block randomization on a 1:1 ratio was used, with 7 blocks formed by sex, age (<50 years or ≥50 years), and profession (physician, nurse, or physical therapist). Randomization to receive CBD plus standard care or standard care alone was performed by an independent researcher who was not directly involved with data collection.

### Participants

**Figure 1.**
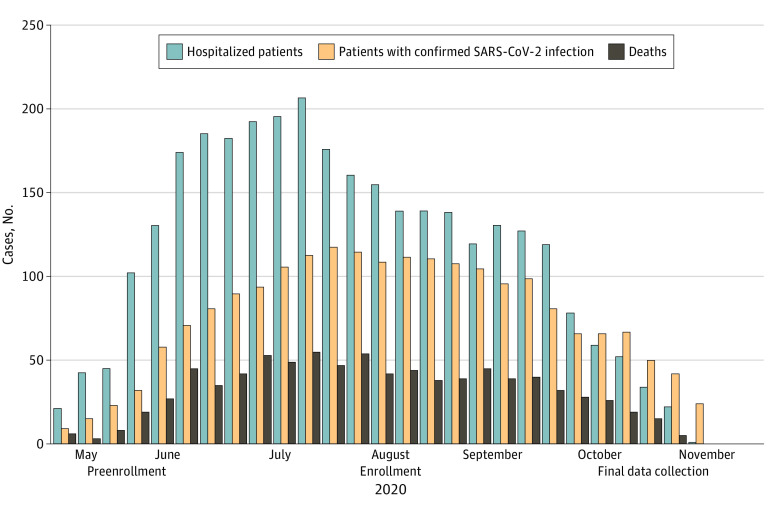
Study Period and First Wave of COVID-19 Cases Period with the start, peak, plateau, and initial reduction curve of the first wave of COVID-19 cases in Ribeirão Preto, São Paulo, Brazil.

Frontline health care professionals (physicians, nurses, and physical therapists) working with patients with COVID-19 at the Ribeirão Preto Medical School University Hospital in São Paulo, Brazil, were recruited. Participants were enrolled between June 12 and November 12, 2020. This period coincided with the start, peak, plateau, and initial reduction curve of the first wave of COVID-19 cases in Ribeirão Preto (eFigure 1 in Supplement 2). Figure 1 summarizes the enrollment and data collection periods.

To recruit participants, the human resources office of the Ribeirão Preto Medical School University Hospital sent an email to its frontline health care workers and their immediate supervisors offering the opportunity to participate in the clinical trial. The study advertisement was also available on the main hospital’s website and social media pages. We recruited and assessed 214 frontline health care workers for eligibility. Of those, 120 participants were randomized, and 118 participants received intervention. Participants were healthy male and female adults (aged 24-60 years) who provided written informed consent after a hospital or online meeting presentation and discussion of the study. Women of fertile age were asked if they were sexually abstinent or using approved contraceptive methods. Participants were excluded if they were currently using any medication that may have had potential interactions with CBD, had a history of adverse or undesirable reactions to CBD or other cannabinoids, belonged to a COVID-19 risk group (eg, had diabetes, hypertension, lung disease, hematological disease, chronic kidney disease, and/or immunosuppression), or were pregnant (including male participants who had a pregnant partner) during the study period.

### Procedure

Participants randomized to the treatment arm received standard care plus oral CBD (99.6% purity; PurMed Global) dissolved in medium-chain triglyceride oil (150 mg/mL) at a daily dose of 300 mg (150 mg twice per day) for 4 weeks. The CBD vials were weighed before they were delivered to the participant and after they were returned at the end of the clinical trial to assess treatment adherence. The control group received standard care alone. Standard care consisted of motivational and instructional videos on low-impact physical exercise plus weekly consultations with psychiatrists who offered psychological support (eg, conversations and spiritual support). Hospital executives also maintained a safe and supportive working environment by repeatedly offering personal protective equipment, modifications to work schedules, and ongoing testing as well as hiring new personnel and providing a specific outpatient treatment unit.

All participants were assessed remotely (via cell phone or computer) using weekly self-rating scales with an estimated completion time of 10 minutes per session. The instruments were completed at baseline and at the end of each of the 4 weeks of the clinical trial. Collected data were recorded in an electronic database by the clinical trial coordination team (of which author J.C.P. was a member).

Treatment safety was assessed using a modified version of the Udvalg for Kliniske Undersøgelser (UKU) Side Effect Rating Scale of the Scandinavian Society of Psychopharmacology^24^; this scale was adapted to create the 15-item CBD Adverse Effects (CARE) scale (score range, 0-45, with 0 indicating no AEs, 1 indicating mild AEs, 2 indicating moderate AEs, and 3 indicating severe AEs).^25^ Participants were also assessed remotely by psychiatrists who evaluated their capacity to manage chronic workplace stress. These assessment sessions included a structured interview to evaluate whether participants fulfilled criteria for the presence of occupational burnout syndrome (eg, low energy or exhaustion, job negativity, and reduced efficiency) according to the *International Classification of Diseases, Eleventh Revision* (*ICD-11*). The psychiatrists were available to provide additional psychological support outside of the weekly assessments if necessary.

Data and observations made by staff were examined by an independent committee that decided whether to continue or suspend treatment according to results on the tolerability and efficacy of the CBD therapy. The monitoring committee was composed of expert clinical researchers who were not associated with the clinical trial. Data were collected by hospital staff, clinical research associates, and clinical trial assistants. Two authors (A.W.Z. and F.S.G.) who were not involved in data collection throughout the clinical trial performed the statistical analyses and interpreted the results, remaining blind to the intervention arms until the study concluded.

### Outcomes

The primary outcome was the efficacy and safety of daily administration of CBD plus standard care vs standard care alone for 4 weeks to reduce or prevent the manifestation of emotional exhaustion and burnout symptoms. Symptom levels were measured using the validated Brazilian version of the emotional exhaustion subscale of the Maslach Burnout Inventory–Human Services Survey for Medical Personnel (MBI-HSS; subscale score range, 0-54 points, with higher scores indicating greater emotional exhaustion).^21^ Secondary outcomes were anxiety, depression, and PTSD symptoms as measured using the validated Brazilian versions of the 7-item Generalized Anxiety Disorder (GAD-7) questionnaire (score range, 0-21 points, with higher scores indicating more severe anxiety),^26^ the 9-item Patient Health Questionnaire (PHQ-9; score range, 0-27 points, with higher scores indicating more severe depression),^27^ and the PTSD Checklist for the *Diagnostic and Statistical Manual of Mental Disorders* (Fifth Edition) (PCL-5; score range, 0-80, with higher scores indicating more severe symptoms of PTSD),^28^ respectively. The Clinical Global Impression scale^29^ was used to assess burnout symptoms, and the daily Ecological Momentary Assessment^30^ was used to assess mood (eMethods 1 in Supplement 2). Psychiatrists who evaluated the criteria for occupational burnout syndrome and rated AEs and results from the Clinical Global Impression scale remained blind to participants’ intervention arms. Blood samples were collected at baseline and days 7, 14, 21, and 28 at the participants’ workplaces to assess proinflammatory cytokine (interleukin 1β [IL-1β] and tumor necrosis factor α [TNF-α]) levels, CBD plasma levels, and general laboratory measurements (eMethods 2 in Supplement 2).

### Statistical Analysis

Because no previous studies had examined the effects of CBD therapy on the reduction of emotional exhaustion among frontline health care professionals working with patients with COVID-19, we calculated sample size by estimating a significance level of α = .05, a statistical power of 0.8, and an effect size (Cohen *f*) of 0.10, resulting in a sample of 114 participants. Collected data were automatically stored in the REDCap platform (Vanderbilt University) and exported to SPSS software, version 26.0 (SPSS Statistics), for analysis. Data from the rating scales were analyzed with a repeated-measures analysis of variance (ANOVA), with factors comprising time, group, and time-group interaction. The degrees of freedom of the repeated factor were corrected using Huynh-Feldt ε when sphericity conditions were not met. Within-subjects contrasts with a significant time-group interaction were used to assess the differences between groups for each measure compared with baseline. Based on a structured interview, participants were evaluated for the presence of burnout syndrome, and their symptoms were classified according to *ICD-11* criteria. The presence of clinical depression or anxiety was defined as a cutoff score of greater than 10 points on the PHQ-9 or GAD-7, respectively.^26,27^ These results, together with data from the UKU and CARE scales, were analyzed using a Fisher exact test. The significance level was set at 2-tailed *P* < .05.

## Results

### Participants

**Figure 2.**
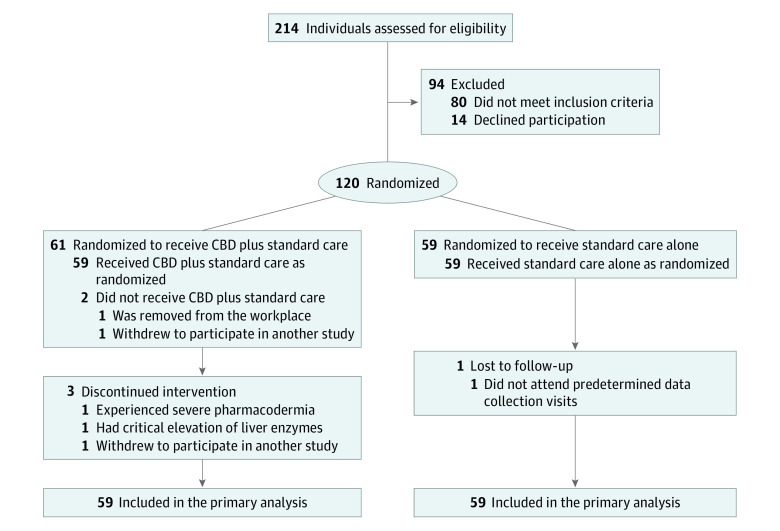
Study Flow Diagram CBD indicates cannabidiol.

Among 214 frontline health care workers assessed for eligibility, 120 participants were randomized (61 to the treatment arm and 59 to the control arm), and 118 participants (79 women [66.9%]; mean age, 33.6 years [95% CI, 32.3-34.9 years]) received intervention as randomized (Figure 2). One individual randomized to the treatment arm withdrew informed consent to participate in another clinical study before starting the intervention. A total of 4 participants (3.4%) discontinued intervention; 3 participants were in the treatment arm (1 experienced severe pharmacodermia, 1 had critical elevation of liver enzymes, and 1 withdrew to participate in the clinical trial of a COVID-19 vaccine), and 1 participant was in the control arm (did not adhere to completion of assessments). The case of severe pharmacodermia was confirmed by serological tests (excluding COVID-19 and other infections), biopsy, and a test based on the CBD formulation. During follow-up, both participants in the treatment arm who discontinued CBD therapy because of serious AEs experienced full recovery. All data collected until discontinuation were included in the analyses. Thus, 118 participants (59 in the treatment arm and 59 in the control arm) were included in the final analysis.

No substantial differences in demographic or clinical characteristics were observed between study arms (Table 1). In both the treatment and control arms, most participants were employed as nurses (32 participants [54.2%] vs 33 participants [55.9%], respectively) in an intensive care unit (26 participants [44.1%] vs 22 participants [37.3%]). In the treatment vs control arms, 21 participants (35.6%) vs 23 participants (39.0%), respectively, had previously received psychological and/or psychiatric treatment; 16 participants (27.1%) vs 10 participants (16.9%) were currently receiving medication for a clinical condition; and 13 participants (22.0%) in each arm currently had a physical illness. Only 8 participants (13.6%) in the treatment arm and 11 participants (18.6%) in the control arm had previously received a positive test result for SARS-CoV-2.

**Table 1.** Demographic and Clinical Characteristics of Participants at Baseline

Characteristic	Participants, No. (%)	
	CBD plus standard care (n = 59)	Standard care alone (n = 59)
Age, mean (95% CI), y	33.4 (31.3-35.3)	33.9 (32.1-35.7)
Sex		
Female	39 (66.1)	40 (67.8)
Male	20 (33.9)	19 (32.2)
BMI, mean (95% CI)	26.6 (25.3-27.9)	26.4 (25.3-27.3)
Occupation		
Physician	25 (42.4)	25 (42.4)
Nurse	32 (54.2)	33 (55.9)
Physical therapist	2 (3.4)	1 (1.7)
Clinical setting		
ICU	26 (44.1)	22 (37.3)
Semi-ICU	9 (15.3)	10 (16.9)
Hospital ward	17 (28.8)	16 (27.1)
Emergency department	7 (11.9)	11 (18.6)
Living situation		
Alone	23 (39.0)	16 (27.1)
With partner and/or children	28 (47.5)	33 (55.9)
With parents	4 (6.8)	8 (13.6)
With friends	4 (6.8)	2 (3.4)
Previous SARS-CoV-2 infection (IgG/IgM positive test result)	8 (13.6)	11 (18.6)
Psychological and/or psychiatric treatment		
Current	16 (27.1)	14 (23.7)
Past	21 (35.6)	23 (39.0)
Physical illness	13 (22.0)	13 (22.0)
Clinical medication	16 (27.1)	10 (16.9)
Psychiatric medication	9 (15.3)	13 (22.0)
Current smoking	10 (16.9)	9 (15.3)
Alcohol use weekly	16 (27.1)	13 (22.0)
Cannabis use	6 (10.2)	5 (8.5)

Abbreviations: BMI, body mass index (calculated as weight in kilograms divided by height in meters squared); CBD, cannabidiol; ICU, intensive care unit; IgG, immunoglobin G; IgM, immunoglobin M.

### Primary Outcome

**Figure 3.**
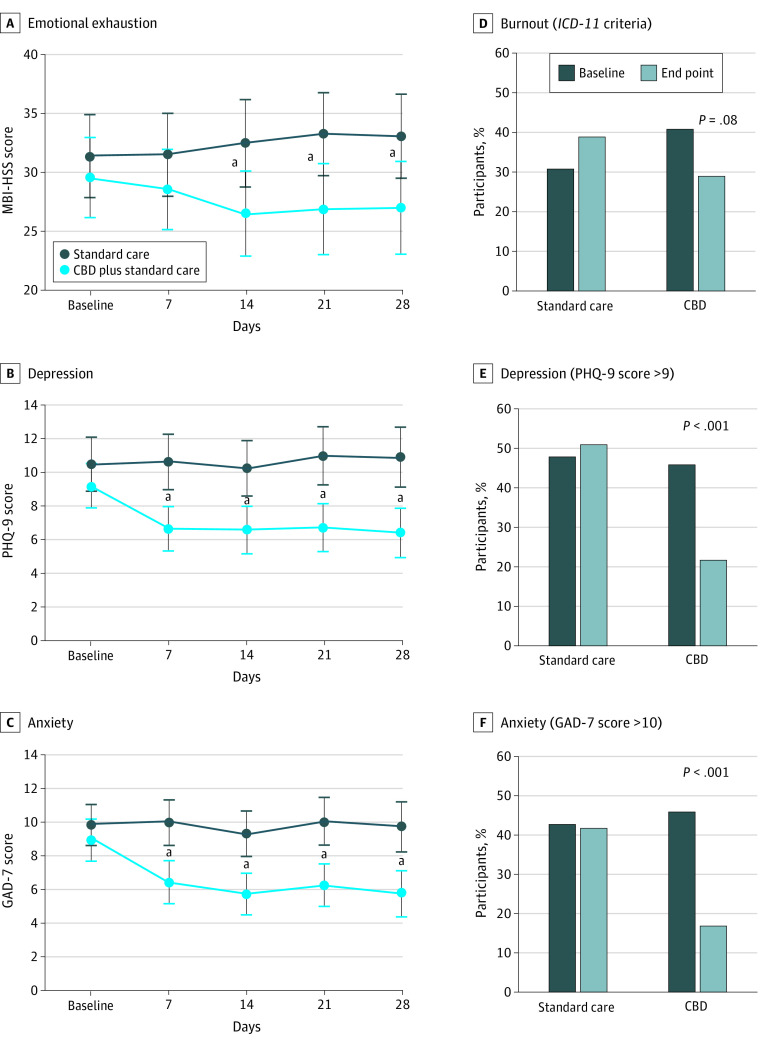
Results for Emotional Exhaustion, Depression, and Anxiety GAD-7 indicates 7-item Generalized Anxiety Disorder questionnaire; *ICD-11*, *International Classification of Diseases, Eleventh Revision*; MBI-HSS, Maslach Burnout Inventory–Human Services Survey for Medical Personnel; and PHQ-9, 9-item Patient Health Questionnaire. ^a^Difference between the treatment and control arms was statistically significant.

With regard to emotional exhaustion, there were no significant effects of time (*F*_2.98-346.10_ = 0.41; *P* = .74) or group (*F*_1.00-116.00_ = 3.75; *P* = .06) on MBI-HSS scores, but a significant time-group interaction (*F*_2.98-346.10_ = 3.83; *P* = .01) was observed (Figure 3). Compared with participants who received standard care alone, participants who received CBD plus standard care had substantial reductions in MBI-HSS scores at day 14 (mean difference, 4.14 points; 95% CI, 1.47-6.80 points; partial eta squared [η_p_^2^] = 0.08) and day 21 (mean difference, 4.34 points; 95% CI, 0.94-7.73 points; η_p_^2^ = 0.05) and a significant reduction in MBI-HSS scores at day 28 (mean difference, 4.01 points; 95% CI, 0.43-7.59 points; η_p_^2^ = 0.04) compared with baseline (eTable 1 in Supplement 2). Symptoms of burnout syndrome (based on *ICD-11* criteria) also decreased between baseline (24 participants [40.7%]) and week 4 (17 participants [28.8%]; *P* = .08) in the treatment group; however, the difference was not statistically significant.

### Secondary Outcomes

With regard to anxiety, there were significant effects of time (*F*_3.35-388.43_ = 6.82; *P* < .001), group (*F*_1.00-116.00_ = 15.34; *P* < .001), and time-group interaction (F_3.35-388.43_ = 5.31; *P* = .001) on GAD-7 scores (Figure 3). Compared with baseline, GAD-7 scores in the treatment group significantly decreased by day 28 (mean difference, 2.79 points; 95% CI, 1.03-4.56 points; η_p_^2^ = 0.08) (eTable 1 in Supplement 2).

For depression, there were significant effects of time (*F*_3.37-390.31_ = 3.83; *P* = .008), group (*F*_1.00-116.00_ = 13.17; *P* < .001), and time-group interaction (*F*_3.37-390.31_ = 5.51; *P* < .001) on PHQ-9 scores (Figure 3). In the treatment group, PHQ-9 scores decreased by day 28 compared with the baseline (mean difference, 2.72 points; 95% CI, 0.95-4.48 points; η_p_^2^ = 0.08) (eTable 1 in Supplement 2). A significant reduction in the number of participants with GAD-7 and PHQ-9 scores higher than the cutoff value (>10 points) at week 4 was observed in the treatment group only.

With respect to PTSD, the difference in baseline PCL-5 scores between the treatment arm (9.8 points) and the control arm (12.4 points) were not significant (mean difference, 2.12 points; 95% CI, −0.15 to 4.40 points; *P* = .06). Therefore, we performed a repeated-measures ANOVA with variations in relation to baseline scores. This analysis did not indicate a significant effect of group (*F*_1.00-116.00_ = 0.03; *P* = .87), time (*F*_2.97-329.65_ = 1.94; *P* = .12), or time-group interaction (*F*_2.97-329.65_ = 0.24; *P* = .87) on PCL-5 scores.

### Cytokine Levels

No difference in IL-1β plasma levels was found between the treatment and control arms at baseline vs week 4. However, IL-1β levels significantly increased in both arms from baseline to week 4 (*F*_1.00-107.00_ = 21.07; *P* < .001). No significant effect on TNF-α plasma levels was observed in either arm (eFigure 2 in Supplement 2).

### CBD Plasma Levels

Plasma levels of CBD, although variable among participants, did not significantly change during the study period, varying between 15.23 ng/mL (interquartile range [IQR], 12.23-30.30 ng/mL) at day 7 and 20.62 ng/mL (IQR, 12.05-27.14 ng/mL; Friedman *P* = .35) at day 21 (eFigure 3 in Supplement 2). No significant associations were observed between CBD plasma levels and score changes on the MBI-HSS, GAD-7, PHQ-9, or PCL-5.

### Safety

The most common AEs (ie, those occurring in >10% of participants) in both arms were somnolence (34 participants [28.8%]), fatigue (27 participants [22.9%]), increased appetite (19 participants [16.1%]), diarrhea (13 participants [11.0%]), weight gain (12 participants [10.2%]), and lethargy (12 participants [10.2%]) (Table 2; eFigure 4 in Supplement 2). The only significant difference between groups was increased appetite among participants in the control arm compared with the treatment arm (14 participants [23.7%] vs 5 participants [8.5%]; *P* = .04). Five serious AEs were observed during the study period, all of which occurred in the treatment group; these events included 4 cases of elevated liver enzymes (>3-fold higher than the upper limit; 1 critical and 3 mild, with the mild elevations reported at the final 28-day assessment) and 1 case of severe pharmacodermia. One mild case of pharmacodermia also occurred in the treatment group. Among participants with substantial elevations in liver enzymes, none experienced a greater than 2-fold increase in normal levels of total serum bilirubin. All participants fully recovered after CBD therapy was discontinued.

**Table 2.** Adverse Events by Treatment Arm

Event	Participants, No. (%)		*P* value^c^
	CBD plus standard care (n = 59)^a^	Standard care alone (n = 59)^b^	
Serious adverse events			
Elevated liver enzymes (>3-fold higher than upper limit)	4 (6.8)	0	.06
Pharmacodermia	1 (1.7)	0	.49
Other adverse events			
Somnolence	15 (25.4)	19 (32.2)	.54
Fatigue	11 (18.6)	16 (27.1)	.38
Diarrhea	6 (10.2)	7 (11.9)	>.99
Sore throat	6 (10.2)	1 (1.7)	.06
Increased appetite	5 (8.5)	14 (23.7)	.04
Headache	4 (6.8)	1 (1.7)	.20
Lethargy	4 (6.8)	8 (13.6)	.36
Weight gain	3 (5.1)	9 (15.3)	.13
Decreased appetite	3 (5.1)	8 (13.6)	.20
Nausea	3 (5.1)	4 (6.8)	>.99
Cough	1 (1.7)	5 (8.5)	.21
Weight loss	1 (1.7)	4 (6.8)	.36
SARS-CoV-2 infection	1 (1.7)	2 (3.4)	>.99
Myalgia	1 (1.7)	4 (6.8)	.36
Vomiting	1 (1.7)	2 (3.4)	>.99
Coryza	1 (1.7)	5 (8.5)	.21
Fever	1 (1.7)	1 (1.7)	>.99
Mild pharmacodermia	1 (1.7)	0	.49
Feeling unwell	1 (1.7)	0	.49
Hyposmia	0	3 (5.1)	.25
Productive cough	0	2 (3.4)	.50
Taste reduction	0	1 (1.7)	>.99

Abbreviation: CBD, cannabidiol.

^a^ Three participants in the treatment arm discontinued intervention (1 had severe pharmacodermia, 1 had critical elevation of liver enzymes, and 1 withdrew to participate in the clinical trial of a COVID-19 vaccine). All data collected until discontinuation were included in the analyses.

^b^ One participant in the control arm discontinued intervention because of nonadherence to completion of assessments.

^c^ *P* values based on the Fisher exact test.

No group-time effect in laboratory measurements was found, with the exception of total cholesterol (*F*_3.80-363.40_ = 4.91; *P* = .001) and low-density lipoprotein cholesterol (*F*_4.00-384.00_ = 4.83; *P* = .001) (eTable 2 in Supplement 2). In the treatment arm, total cholesterol increased on day 21 (mean difference, 8.43 mg/dL; 95% CI, 1.54-15.32 mg/dL; *P* = .009 [to convert to to millimoles per liter, mutliply by 0.0259]) and day 28 (mean difference, 8.32 mg/dL; 95% CI, 0.66-15.98 mg/dL; *P* = .03) compared with day 7 and on day 21 compared with day 14 (mean difference, 6.37 mg/dL; 95% CI, 0.92-11.83 mg/dL; *P* = .02). Low-density lipoprotein cholesterol also increased on day 21 compared with day 7 (mean difference, 7.67 mg/dL; 95% CI, 1.89-13.45 mg/dL; *P* = .003 [to convert to to millimoles per liter, mutliply by 0.0259]) and day 14 (mean difference, 5.76 mg/dL; 95% CI, 0.67-10.84 mg/dL; *P* = .02). No significant differences in these 2 variables were found between groups at any point during the study period.

## Discussion

This randomized clinical trial found that the efficacy and safety of daily treatment with CBD, 300 mg, for 4 weeks combined with standard care was superior to standard care alone for reducing the symptoms of emotional exhaustion, anxiety, and depression among frontline health care professionals working with patients with COVID-19. Furthermore, treatment with CBD plus standard care decreased the number of diagnoses of burnout syndrome (based on *ICD-11* criteria) and significantly reduced the number of participants with scores indicative of anxiety (GAD-7 score >9 points) and depression (PHQ-9 score >9 points) at 4 weeks after initiation of treatment. Treatment with CBD was associated with mostly mild and transient AEs, similar to those reported in the control arm. The few reported cases of serious AEs resolved after discontinuation of CBD, but their presence highlights the need for close clinical monitoring (especially liver function testing) of patients receiving CBD therapy.^10^

Burnout among health care workers is an important issue for health care systems, with a direct impact on quality of care. No pharmacological treatment is currently available for the prevention or treatment of burnout symptoms and emotional exhaustion among frontline health care professionals working with patients with COVID-19, even though several studies have reported that depression, anxiety, insomnia, and PTSD symptoms are more common in this population.^2,6^ Therefore, the results of the present study could have a relevant impact on the mental health of health care staff worldwide. Cannabidiol has been found to have anxiolytic effects in humans^8,9,10,11^ at doses similar to those used in the present clinical trial. Our results are also consistent with preclinical evidence suggesting that CBD has antidepressant properties.^12,13,14^

Burnout syndrome,^31^ depression,^32^ and stress-related disorders^33^ appear to be associated with higher levels of proinflammatory cytokines.^31^ Increased levels of IL-1β could help to identify patients with treatment-resistant depression.^34^ Our results suggest that this cytokine may also be involved in burnout syndrome. However, although CBD has been reported to have anti-inflammatory effects,^17,18,19,20^ the drug did not prevent increases in IL-1β levels among participants in the current study. Additional clinical trials are necessary to investigate the role of inflammatory mediators in the beneficial effects of CBD on mental health.

### Limitations

This study has limitations. These limitations include the study’s lack of a double-blind placebo-controlled design, short follow-up period, single-intervention dose, and single-center design. Therefore, caution should be applied in generalizing these findings to participants from other regions or in different contexts. We also did not assess cognition using neuropsychological tests. However, CBD has not been reported to decrease cognitive performance in healthy adults,^8^ making it unlikely that the present findings are associated with drug-induced cognitive impairment.

Given that participants and investigators in the present clinical trial were not blinded, any difference that favored the treatment arm could have come from 1 or more of 3 sources: (1) an effect of CBD, (2) a placebo effect, and/or (3) biased outcome reporting because nonblinded participants self-reported their outcomes. However, all investigators responsible for data collection and analysis were blinded with respect to study arms, and our design was similar to that of previous investigations comparing standard care with pharmacological interventions among patients with COVID-19. In addition, the sample size ensured robust statistical power to assess a population experiencing high levels of stress given that data were collected during the worst period of the first wave of the pandemic in our catchment area and among participants with an increased incidence of clinical symptoms.

## Conclusions

Daily administration of CBD, 300 mg, combined with standard care reduced the symptoms and diagnoses of anxiety, depression, and emotional exhaustion among frontline health care professionals working with patients with COVID-19. Cannabidiol may act as an effective agent for the reduction of burnout symptoms among a population with important mental health needs worldwide. However, it is necessary to balance the benefits with potential adverse and undesired effects when making decisions regarding the use of this compound.^10^ Future double-blind placebo-controlled clinical trials are needed to assess whether the conclusions drawn from the present study can be more broadly applied.

## Notes

Supplement 1.
Trial Protocol

Supplement 2. **eMethods 1.** Subjective Scales **eMethods 2.** Blood Tests, Quantification of Proinflammatory Cytokines, and Analysis of Plasma Levels of CBD **eTable 1.** Score Differences Compared With Baseline **eTable 2.** Laboratory Measurements **eFigure 1.** Catchment Area of Ribeirão Preto, São Paulo State Region in the Health Regional Departments of São Paulo, Brazil **eFigure 2.** Cytokine Plasma Levels (IL-1β and TNF-α) Between Study Arms (Baseline vs Week 4) **eFigure 3.** CBD Plasma Levels at Time Points During Study **eFigure 4.** Most Common (>10%) Adverse Events in Study Arms
eReferences

Supplement 3.
Nonauthor Collaborators

Supplement 4.
Data Sharing Statement
