# Supplementary material for: Efficacy and Safety of Cannabidiol Plus Standard Care vs Standard Care Alone for the Treatment of Emotional Exhaustion and Burnout Among Frontline Health Care Workers During the COVID-19 Pandemic: A Randomized Clinical Trial
Source: JAMA Netw Open. 2021 Aug 13;4(8):e2120603. doi: 10.1001/jamanetworkopen.2021.20603 (PMC8363917; doi:10.1001/jamanetworkopen.2021.20603)
Supplement: Supplement 1. — Trial Protocol [file jamanetwopen-e2120603-s001.pdf]

Supplement

1. Trial protocol

PROTOCOL VERSION 4.2, 05/27/21
ClinicalTrials.gov Identifier: NCT04504877

Project Title:
Burnout and Distress preventiOn With caNnabidiol in Front-line Health CareworkerS deAling wIth COVID-19
Short title: BONSAI Study

Principal Investigator:
Prof José Alexandre de Souza Crippa - Full Professor - Psychiatry, University of São Paulo

Co-Investigators:
Prof Antonio W. Zuardi
Prof Francisco S. Guimarães
Prof Flávia de Lima Osório
Prof Alline Cristina Campos
Prof Sonia Regina Loureiro
Prof Rafael Guimarães dos Santos
Dr José Diogo dos Santos Souza
Dr Juliana Mayumi
Julia Cozar Pacheco
Prof Raphael Mechoulam
Prof Flávio Kapczinski
Prof Benedito AL Fonseca
Danillo LA Esposito
Karina Pereira-Lima
Prof Srijan Sem
Maristela Haddad Andraus
Prof Jaime E. C. Hallak

Main study centres
Department of Neuroscience and Behavior
Division of Psychatry
Ribeirão Preto Medical School
University of São Paulo
Ribeirão Preto
Brazil
14049-900

Department of Pharmacology
Ribeirão Preto Medical School
University of São Paulo
Ribeirão Preto, Brazil

The Institute for Drug Research
School of Pharmacy
The Hebrew University of Jerusalem
Jerusalem, Israel.

Department of Internal Medicine
Ribeirão Preto Medical School
University of São Paulo

Ribeirão Preto, Brazil.

Department of Psychiatry
University of Michigan Medical School
Ann Arbor, Michigan

Department of Psychiatry
Federal University of São Paulo
São Paulo, Brazil.

Chromatox Laboratory Ltda
São Paulo, Brazil

Study sponsors
University of São Paulo

Financial support
The São Paulo Research Foundation
National Institute for Science and Technology – Translational Medicine, Brazil

1.1 Aim of study

Our primary aim is to investigate the safety and efficacy of Cannabidiol (CBD) as an intervention in reducing burnout symptoms in a group of front-line health professionals working with COVID-19 patients at the Ribeirão Preto Medical School University Hospital (RPMSUH) for four weeks.

Secondary aims are:

- Assess the level of stress and emotional overload of front-line health workers (physicians, nurses, and physiotherapists) during their performance in the pandemic caused by COVID-19.
- Assess whether the daily use of CBD 300 mg, for four weeks, reduces the level of stress, during the period of performance of professionals in the care of patients withCOVID-19.
- Assess whether the daily use of CBD 300 mg, for four weeks, will modify inflammatory parameters, such as cytokines, measured from the serum of professionals in the care of patients with COVID-19.
- Assess whether the daily use of CBD 300 mg, for four weeks, prevents depression, burnout and Acute Stress Disorder and PTSD.
- Assess the possible adverse effects of using CBD.

1.2 Background of the study

The severe acute respiratory syndrome coronavirus 2 (SARS-CoV-2 or COVID-19) outbreak started in the Wuhan province in China in late December 2019. The World Health Organization (WHO) declared the disease as a pandemic on March 11, 2020. Since then, the pandemic has claimed more than two million lives worldwide. In this scenario, most countries adopted lockdown and physical distancing as containment strategies. This situation led to social isolation, decreased family and social support, and loneliness, which affected several aspects of our daily lives, including mental health.[1-4] Social isolation measures can induce or exacerbate mental health disorders since they are often associated with income loss, poverty, and limited access to essential services.

Since February 2020, several nationwide, large-scale surveys in the general population during the pandemic showed increases in moderate to severe emotional distress symptoms, depression, and anxiety,[3-5-9] post-traumatic stress disorder (PTSD),[3,4,6,7,9,10] and insomnia.[3,4,7] Depression, anxiety, insomnia, and emotional distress are especially prevalent among healthcare workers dealing with COVID-19 patients, such as physicians and nurses.[2-5,11] A recent meta-analysis of 13 cross-sectional studies (12 from China, one from Singapore; N = 33062) assessing the mental health of healthcare workers estimated prevalence rates of 23.2% of anxiety, 38.9% of insomnia, and 22.8% of depression symptoms within this population. [11]

This gloomy scenario increases individual and social challenges and is expected to be accompanied by increases in other mental health problems, such as substance use disorders and suicide attempts.[3,4] Symptoms of emotional distress, depression, anxiety, and insomnia are usually treated with traditional antidepressants, anxiolytics, and hypnotics. However, these drugs often need several weeks to produce their therapeutic effects and have significant adverse effects. A safer and more effective drug to treat and prevent the manifestation and/or exacerbation of anxiety, depression, and other emotional exhaustion symptoms is highly desirable, especially during the current COVID-19 pandemic.

Cannabidiol (CBD) is a non-psychotomimetic phytocannabinoid with favorable safety and tolerability profiles shown to have anxiolytic effects in controlled trials with healthy volunteers[12-15] and adults[16,17] and teenagers[18] with social anxiety disorder. CBD doses in those studies ranged from single doses of 300–600 mg[12-16] to 300 mg daily for four weeks.[18] There is also evidence of antidepressant effects of CBD in preclinical studies.[19,20] Furthermore, preclinical studies showed that CBD has anti-inflammatory[21-23] and antiviral[24] properties. Indeed, CBD reversed the cytokine storm (reduced levels of interleukin-6, IL-6; tumor necrosis factor-α, TNFα; and interferon-γ, IFNγ expression) and symptoms of acute respiratory distress syndrome (ARDS) very similar to those of severe COVID-19 in mice.[21-22] Moreover, CBD decreased the levels of pro-inflammatory cytokines (IL-4, IL-5, IL-6, IL-13) in a mice model of allergic asthma.[23] Besides the possible benefits of CBD on the viral load and cytokine storm induced by COVID-19, there is an association between distress/anxiety/depression and peripheral and central inflammatory processes. Thus, considering the anxiolytic, antidepressant, and anti-inflammatory properties of CBD and its good safety profile, we designed and conducted a single-site trial assessing the efficacy and safety of 300 mg CBD twice a day for four weeks in reducing symptoms of anxiety, depression, and emotional distress in front-line healthcare workers (physicians, nurses, and physical therapists) treating COVID-19 patients in a Brazilian hospital.

2. Methods

2.1 Design

The study will be a single-site, two-arm, parallel-group, participant and investigator unblinded, evaluator blinded, superiority trial of oral CBD 300 mg daily for 28 days to evaluate prevention or reduction of emotional distress in healthcare workers dealing with COVID-19 patients. After 28 days, the trial will include an open-label extension period where all participants will be offer treatment with CBD 300 mg for the same period of the original trial (28 days). Front-line healthcare workers (physicians, nurses, and physical therapists) working with COVID-19 patients at the Ribeirão Preto Medical School University Hospital (RPMSUH) will be invited to participate in the study. Volunteers will be randomized by block randomization with a 1:1 allocation ratio by a researcher who will not directly involved with data collection to receive CBD for four weeks. This study will have two arms (described in table 1):

1. CBD 300 mg/daily plus General measures (supporting motivational videos, fitness videos);
2. General measures (supporting motivational videos, fitness videos) alone.

This study will follow the guidelines of the Consolidated Standards of Reporting Trials (CONSORT) statement.

ARM	INTERVENTION / TREATMENT
**Experimental: Cannabidiol plus general clinical supportive measures.**<br><br>The participants will receive CBD 300 mg/daily plus general measures (supporting motivational videos, fitness videos). All the participants will perform invasive and non-invasive procedures with the nursing team, the invasive being five blood collections, in their workplaces, to evaluate laboratory parameters, detailed in this project. The non-invasive ones refer to the measurement of height, weight, body temperature, and sleep pattern, as well as five collections of saliva, in a collecting tube, to assess viral load. They will also be monitored through	Drug: Cannabidiol<br>Cannabidiol 300 mg daily for 4 weeks<br>+<br>General clinical supportive measures<br>(days 0, 7, 14, 21, and 28)

weekly self-assessment scales, applied remotely, electronically (cell phones or computers), with an estimated duration of ten minutes each, which will have their responses recorded in an electronic database by the study coordination team.	
**General clinical supportive measures**<br><br>The participants will receive general measures (supporting motivational videos, fitness videos) alone. All the participants will perform invasive and non-invasive procedures with the nursing team, the invasive being five blood collections, in their workplaces, to evaluate laboratory parameters, detailed in this project. The non-invasive ones refer to the measurement of height, weight, body temperature, and sleep pattern in a collecting tube to assess viral load. Also, they will be monitored through weekly self-assessment scales, applied remotely, electronically (cellphones or computers), with an estimated duration of ten minutes each, which will have their responses recorded in an electronic database by the study coordination team.	General clinical supportive measures<br>(days 0, 7, 14, 21, and 28)

Table 1: Study description - Groups and interventions.

2.2 Volunteers

Sample size: 120 patients
Males & females
24 to 60 years old

120 Front-line healthcare workers (physicians, nurses, and physical therapists) working with COVID-19 patients at the Ribeirão Preto Medical School University Hospital (RPMSUH) will be invited to participate in the study.

An initial meeting will be held at RPMSUH, for the presentation and discussion of the project, answering the doubts of eventual participants who will have the option to participate, considering the inclusion and exclusion criteria. Those who agree, and are able to participate in the study, will be invited to sign the Informed Consent Form.

After signing the informed consent form, during the study, the research participants will perform invasive and non-invasive procedures, with the invasive 5 blood samples being taken from their workplaces to assess laboratory parameters, detailed in this project. The non-invasive ones refer to the measurement of height, weight, body temperature, and sleep pattern, as well as five collections of saliva, in a collecting tube, to assess viral load. In addition to being monitored through weekly self-assessment scales, applied remotely, electronically (cell phones or computers), with an estimated duration of ten minutes each, which will have their responses recorded in an electronic database, by the coordination team of the study, and analyzed, for the compilation of data and evaluation of CBD efficacy and safety, by the physicians responsible for the study.

Those who are in the study arm which refers to the use of CBD receive the investigational product, at four weeks. The use of CBD at home will be guided by the pharmacy team of the research project. After the four-week period, if the CBD proves to be effective and well tolerated, there will be an expansion phase of the study, where the possibility of using cannabidiol will be offered to the other half of the participants. At the end of the fourth week of the study, after analyzing the results, the medical team together with each participant will decide whether or not to continue using the CBD.

If the product under investigation does NOT show tolerability and effectiveness, after the initial four weeks, the pharmacy team will collect the investigational product from the research participants, whether they are expanding or not, and the use of CBD will be suspended.

2.2.1 Inclusion criteria

1) Age between 24 and 60 years old;

2) Both sexes;

3) HCFMRP physicians, nurses and physiotherapists involved in the care of patients who have undergone screening and present at least one symptom, which may be related to COVID-19 infection.

4) Good health conditions and without conditions that characterize them as belonging to the risk groups associated with COVID infection19.

5) Research participants with the potential to become pregnant may be included in the study if they are sexually abstinent or using a contraceptive method considered effective.

6) Signing of the Free and Informed Consent Form (ICF) by the Research Ethics Committee (CEP) and CONEP.

2.2.2 Exclusion criteria

1) Using any medication with potential interaction with CBD or with a history of undesirable reactions prior to the use of this cannabinoid.

2) Physicians, nurses and physiotherapists with occasional contact with patients with COVID-19 and who are not responsible for the continuous monitoring of these patients.

3) Although in the RPMSUH care allocation policy, professionals who are eventually more susceptible to complications from the new coronavirus will be relocated to not attend patients with COVID19, it will be checked again to exclude patients with chronic diseases: diabetes, hypertension, lung disease like asthma or COPD; hematological diseases, chronic kidney disease and immunodepression.

4) Professionals over 60 years old

5) Female research participants who become pregnant or male participants who have their pregnant partner during the research project

2.3 Outcome Measures

<u>Primary Outcome Measure:</u>

1. MBI-EE: Abbreviated Maslach Burnout Inventory - Human Services Survey [Time Frame: Through study completion, over time during the study period (day 0-28)]

To assess the emotional exhaustion dimension of the burnout syndrome, based onnine items, scored from 0 ("never") to 6 ("every day")

<u>Secondary Outcome Measures:</u>

1. Brief measure for assessing generalized anxiety disorder: The GAD-7 [Time Frame: Through study completion, over time during the study period (day 0-28)]

Brief measure for assessing generalized anxiety disorder.

2. PHQ-9: Patient´s Health Questionnaire-9 [Time Frame: Through study completion, over time during the study period (day 0-28)]

Evaluate depressive symptoms o

3. Change in proinflammatory cytokine concentration [Time Frame: Through study completion, over time during the study period (day 0-28)]

Laboratory parameters, including the change in proinflammatory cytokine concentrations.

4. Number of participants with treatment-related adverse events as assessed by CTCAEv5.0 [Time Frame: Through study completion, over time during the study period (day 0-28)]

Occurrence of side effects

5. Posttraumatic Stress Disorder Checklist for DSM-5 (PCL-5) [Time Frame: Through study completion, over time during the study period (day 0-28)]

It is a self-report measure widely used to assess PTSD symptoms, according to theDSM-5 criteria. The reduced version of this instrument will be used (8 items)

6. Samples for blood tests

- Full hemogram;
- Sodium/potassium;
- Urea;

- Creatinine;
- Total bilirubin;
- Oxalacetic transaminase;
- Pyruvic transaminase;
- Gamma-glutamyltransferase;
- Alkaline phosphatase;
- Glucose;
- Lipidogram;
- Thyroid-stimulating hormone;
- Testosterone;
- Cortisol;
- Estrogen;
- Progesterone;
- Proinflammatory cytokines (IL-10, TNFα)
- Plasma levels of CBD.

3. Drug

Cannabidiol 99.6% purity, supplied by PurMed Global (Palm Beach, Florida, USA), dissolved in medium-chain triglyceride (MCT) oil with a concentration of 150 mg/mL. Daily administration will be 300 mg CBD (1 mL or 150 mg per dose, twice a day) for four weeks. The bottles containing the product under investigation will be weighed before delivery to the research participant and at the time of returning the bottles, which must be retained with the research participant, so that it is possible to calculate adherence to the pharmacological treatment and registration in form.

4. Analysis of results

The collected data will be automatically archived on the platform and then exported to the SSPS Software for analysis.

The clinical and demographic data of the participants, who did or did not use CBD, will be compared using the T test for continuous data and the chi-square test for nominal data.

The total scores of the scales: MBI-EE, PCL-5, PHQ-9, GAD-7, CGI-S, will be subjected to Analysis of Variance of repeated measures (ANOVAmr). The data from the UKU / CARE scale and laboratory tests will be presented descriptively.

The statistical analysis will be performed with the aid of the Statistical Package for the Social Sciences (SPSS) v.22.0 software produced by Statistical Product and Service Solutions Incorporation (www.spss.com.br).

5. Ethical aspects

Health professionals assigned to care for patients with COVID-19 will be invited to participate in the research, this participation being absolutely voluntary. The subject will be assured at the time of the invitation, that there will be complete freedom to withdraw their consent at any time during the course of the research and to stop participating in the study without causing any harm.

All volunteers will have access to the consent form and the clarification of all their doubts. Only subjects who sign the free and informed consent form will be accepted in the study. The right to receive information and clarifications regarding any questions that arise during the procedure and updated information about the study will be guaranteed, even though this may affect your willingness to continue participating. Similarly, the security of not being identified will be guaranteed, as well as that all information provided by them will be kept confidential.

The cannabinoid substance to be used (CBD) is devoid of the typical psychological effects of Cannabis sativa.[25] CBD is a safe compound when administered to humans in a wide dosage range, both in acute administration, orally and in chronic administration[26, 27].

The CBD had its classification changed by Brazilian Health Regulatory Agency (Anvisa), changing from a banned drug to a drug for controlled use. In December 2014, the Federal Council of Medicine authorized the prescription of CBD, in special situations. In human research, this compound has been extensively tested.

6. Schedule

Activities	April/20	May/20	Jun/20	Jul/20	Aug/20	Sep/20	Oct/20	Nov/20	Dec/20	Jan/21
Submission of the Protocol and documents to ethical entities	x									
Sending medication to the Center	x									
Training of the Center involved in the Protocol	x									
Patient Recruitment and Selection	x	x	x	x	x	x				
Conducting the Clinical Trial	x	x	x	x	x	x	x	x		
Preparation of Final Reports	x	x	x	x	x	x	x	x	x	x
Preparation of the scientific article	x	x	x	x	x	x	x	x	x	x

7. Added value of this research

To our knowledge, this is the first investigation of the safety and efficacy of CBD as a prevention treatment for burnout symptoms and other mental health problems in front-line health professionals treating COVID-19 patients.

8. Implications of all the available evidence

The results of this study may collaborate to prevent enormous emotional distress and psychological and mental health consequences already observed in professionals who work in chronic and continuous stress situations, now in particular in the context of the current Pandemic by CoronaVirus-19. Since these professionals are a precious group in the treatment of the population, if these potential individual benefits are observed, we imagine that this will be extended indirectly to social aspects and, consequently, to the health of the whole society.

9. References

1. Brooks SK, Webster RK, Smith LE, et al. The psychological impact of quarantine and how to reduce it: rapid review of the evidence. *Lancet*. 2020;395(10227):912-920. doi:10.1016/S0140-6736(20)30460-8
2. Xiang YT, Yang Y, Li W, et al. Timely mental health care for the 2019 novel coronavirus outbreak is urgently needed. *Lancet Psychiatry*. 2020;7(3):228-229. doi:10.1016/S2215-0366(20)30046-8
3. Moreno C, Wykes T, Galderisi S, et al. How mental health care should change as a consequence of the COVID-19 pandemic. *Lancet Psychiatry*. 2020;7(9):813-824. doi:10.1016/S2215-0366(20)30307-2
4. Simon NM, Saxe GN, Marmar CR. Mental Health Disorders Related to COVID-19-Related Deaths. *JAMA*. 2020;324(15):1493-1494. doi:10.1001/jama.2020.19632
5. Iob E, Frank P, Steptoe A, Fancourt D. Levels of Severity of Depressive Symptoms Among At-Risk Groups in the UK During the COVID-19 Pandemic. *JAMA Netw Open*. 2020;3(10):e2026064. Published 2020 Oct 1. doi:10.1001/jamanetworkopen.2020.26064
6. González-Sanguino C, Ausín B, Castellanos MÁ, et al. Mental health consequences during the initial stage of the 2020 Coronavirus pandemic (COVID-19) in Spain. *Brain Behav Immun*. 2020;87:172-176. doi:10.1016/j.bbi.2020.05.040
7. Ren Y, Zhou Y, Qian W, et al. Letter to the Editor "A longitudinal study on the mental health of general population during the COVID-19 epidemic in China". *Brain Behav Immun*. 2020;87:132-133. doi:10.1016/j.bbi.2020.05.004
8. Qiu J, Shen B, Zhao M, Wang Z, Xie B, Xu Y. A nationwide survey of psychological distress among Chinese people in the COVID-19 epidemic: implications and policy recommendations [published correction appears in Gen Psychiatr. 2020 Apr 27;33(2):e100213corr1]. *Gen Psychiatr*. 2020;33(2):e100213. Published 2020 Mar 6. doi:10.1136/gpsych-2020-100213
9. Wang C, Pan R, Wan X, et al. Immediate Psychological Responses and Associated Factors during the Initial Stage of the 2019 Coronavirus Disease (COVID-19) Epidemic among the General Population in China. *Int J Environ Res Public Health*. 2020;17(5):1729. Published 2020 Mar 6. doi:10.3390/ijerph17051729
10. Tang W, Hu T, Hu B, et al. Prevalence and correlates of PTSD and depressive symptoms one month after the outbreak of the COVID-19 epidemic in a sample of home-quarantined Chinese university students. *J Affect Disord*. 2020;274:1-7. doi:10.1016/j.jad.2020.05.009
11. Pappa S, Ntella V, Giannakas T, Giannakoulis VG, Papoutsi E, Katsaounou P. Prevalence of depression, anxiety, and insomnia among healthcare workers during the COVID-19 pandemic: A systematic review and meta-analysis [published correction appears in Brain Behav Immun. 2021 Feb;92:245]. *Brain Behav Immun*. 2020;88:901-907. doi:10.1016/j.bbi.2020.05.026
12. Zuardi AW, Cosme RA, Graeff FG, Guimarães FS. Effects of ipsapirone and cannabidiol on human experimental anxiety. *J Psychopharmacol*. 1993;7(1 Suppl):82-88. doi:10.1177/026988119300700112
13. Crippa JA, Zuardi AW, Garrido GE, et al. Effects of cannabidiol (CBD) on regional cerebral blood flow. *Neuropsychopharmacology*. 2004;29(2):417-426. doi:10.1038/sj.npp.1300340
14. Zuardi AW, Rodrigues NP, Silva AL, et al. Inverted U-Shaped Dose-Response Curve of the Anxiolytic Effect of Cannabidiol during Public Speaking in Real Life. *Front Pharmacol*. 2017;8:259. Published 2017 May 11. doi:10.3389/fphar.2017.00259
15. Linares IM, Zuardi AW, Pereira LC, et al. Cannabidiol presents an inverted U-shaped dose-response curve in a simulated public speaking test. *Braz J Psychiatry*. 2019;41(1):9-14. doi:10.1590/1516-4446-2017-0015

16. Crippa JA, Derenusson GN, Ferrari TB, et al. Neural basis of anxiolytic effects of cannabidiol (CBD) in generalized social anxiety disorder: a preliminary report. *J Psychopharmacol*. 2011;25(1):121-130. doi:10.1177/0269881110379283
17. Bergamaschi MM, Queiroz RH, Chagas MH, et al. Cannabidiol reduces the anxiety induced by simulated public speaking in treatment-naïve social phobia patients. *Neuropsychopharmacology*. 2011;36(6):1219-1226. doi:10.1038/npp.2011.6
18. Masataka N. Anxiolytic Effects of Repeated Cannabidiol Treatment in Teenagers With Social Anxiety Disorders. *Front Psychol*. 2019;10:2466. Published 2019 Nov 8. doi:10.3389/fpsyg.2019.02466
19. Zanelati TV, Biojone C, Moreira FA, Guimarães FS, Joca SR. Antidepressant-like effects of cannabidiol in mice: possible involvement of 5-HT1A receptors. *Br J Pharmacol*. 2010;159(1):122-128. doi:10.1111/j.1476-5381.2009.00521.x
20. Silote GP, Sartim A, Sales A, et al. Emerging evidence for the antidepressant effect of cannabidiol and the underlying molecular mechanisms. *J Chem Neuroanat*. 2019;98:104-116. doi:10.1016/j.jchemneu.2019.04.006
21. Khodadadi H, Salles ÉL, Jarrahi A, et al. Cannabidiol Modulates Cytokine Storm in Acute Respiratory Distress Syndrome Induced by Simulated Viral Infection Using Synthetic RNA. *Cannabis Cannabinoid Res*. 2020;5(3):197-201. Published 2020 Sep 2. doi:10.1089/can.2020.0043
22. Salles ÉL, Khodadadi H, Jarrahi A, et al. Cannabidiol (CBD) modulation of apelin in acute respiratory distress syndrome. *J Cell Mol Med*. 2020;24(21):12869-12872. doi:10.1111/jcmm.15883
23. Vuolo F, Abreu SC, Michels M, et al. Cannabidiol reduces airway inflammation and fibrosis in experimental allergic asthma. *Eur J Pharmacol*. 2019;843:251-259. doi:10.1016/j.ejphar.2018.11.029
24. Mabou Tagne A, Pacchetti B, Sodergren M, Cosentino M, Marino F. Cannabidiol for Viral Diseases: Hype or Hope?. *Cannabis Cannabinoid Res*. 2020;5(2):121-131. Published 2020 Jun 5. doi:10.1089/can.2019.0060
25. Zuardi AW, Shirakawa I, Finkelfarb E, Karniol IG. Action of cannabidiol on the anxiety and other effects produced by delta 9-THC in normal subjects. *Psychopharmacology (Berl)*. 1982;76(3):245-250. doi:10.1007/BF00432554
26. Bergamaschi MM, Queiroz RH, Zuardi AW, Crippa JA. Safety and side effects of cannabidiol, a Cannabis sativa constituent. *Curr Drug Saf*. 2011;6(4):237-249. doi:10.2174/157488611798280924
27. Iffland K, Grotenhermen F. An Update on Safety and Side Effects of Cannabidiol: A Review of Clinical Data and Relevant Animal Studies. *Cannabis Cannabinoid Res*. 2017;2(1):139-154. Published 2017 Jun 1. doi:10.1089/can.2016.0034
