# Supplementary material for: Efficacy and Safety of Cannabidiol Plus Standard Care vs Standard Care Alone for the Treatment of Emotional Exhaustion and Burnout Among Frontline Health Care Workers During the COVID-19 Pandemic: A Randomized Clinical Trial
Source: JAMA Netw Open. 2021 Aug 13;4(8):e2120603. doi: 10.1001/jamanetworkopen.2021.20603 (PMC8363917; doi:10.1001/jamanetworkopen.2021.20603)
Supplement: Supplement 2. — eMethods 1. Subjective Scales eMethods 2. Blood Tests, Quantification of Proinflammatory Cytokines, and Analysis of Plasma Levels of CBD eTable 1. Score Differences Compared With Baseline eTable 2. Laboratory Measurements eFigure 1. Catchment Area of Ribeirão Preto, São Paulo State Region in the Health Regional Departments of São Paulo, Brazil eFigure 2. Cytokine Plasma Levels (IL-1β and TNF-α) Between Study Arms (Baseline vs Week 4) eFigure 3. CBD Plasma Levels at Time Points During Study eFigure 4. Most Common (>10%) Adverse Events in Study Arms eReferences [file jamanetwopen-e2120603-s002.pdf]

# Supplementary Online Content

Crippa JAS, Zuardi AW, Guimarães FS, et al; Burnout and Distress Prevention With Cannabidiol in Front-line Health Care Workers Dealing With COVID-19 (BONSAI) Trial Investigators. Efficacy and safety of cannabidiol plus standard care vs standard care alone for the treatment of emotional exhaustion and burnout among fronline health care workers during the COVID-19 pandemic: a randomized clinical trial. *JAMA Netw Open*. 2021;4(8):e2120603. doi:10.1001/jamanetworkopen.2021.20603

**eMethods 1.** Subjective Scales

**eMethods 2.** Blood Tests, Quantification of Proinflammatory Cytokines, and Analysis of Plasma Levels of CBD

**eTable 1.** Score Differences Compared With Baseline

**eTable 2.** Laboratory Measurements

**eFigure 1.** Catchment Area of Ribeirão Preto, São Paulo State Region in the Health Regional Departments of São Paulo, Brazil

**eFigure 2.** Cytokine Plasma Levels (IL-1β and TNF-α) Between Study Arms (Baseline vs Week 4)

**eFigure 3.** CBD Plasma Levels at Time Points During Study

**eFigure 4.** Most Common (>10%) Adverse Events in Study Arms

**eReferences**

This supplementary material has been provided by the authors to give readers additional information about their work.

© 2021 Crippa JAS et al. *JAMA Network Open*.

**eMethods 1.** Subjective Scales

Burnout symptom levels were measured with the validated Brazilian version of the emotional exhaustion subscale of the Maslach Burnout Inventory - Human Services Survey (MBI-EE).[1] The MBI-EE is a self-report instrument that assesses the emotional exhaustion dimension in burnout syndrome. It consists of nine items scored from 0 ("*Never*") to 6 ("*Every day*"). Secondary outcome measures included instruments to assess stress levels, anxiety, depression, and PTSD symptoms, and blood tests. Anxiety symptoms were measured with the validated Brazilian version of the Generalized Anxiety Disorder Questionnaire – 7 (GAD-7).[2] The GAD-7 is a self-report instrument that assesses anxiety symptoms through seven items scored from 0 ("*Never*") to 3 ("*Almost every day*"). Scores above 9 points suggest a diagnosis of GAD. Depressive symptoms were measured with the validated Brazilian version of the Patient Health Questionnaire – 9 (PHQ-9).[3] The PHQ-9 is a self-report instrument that measures depressive symptoms through seven items scored from 0 ("*Never*") to 3 ("*Almost every day*"). Scores above 9 points indicate the presence of depressive disorders. PTSD symptoms were measured with the validated Brazilian reduced version of the Posttraumatic Stress Disorder Checklist – 5 (PCL-5).[4] The PCL-5 is a self-report instrument widely used to assess PTSD symptoms according to the DSM-5 criteria through eight items. The Clinical Global Impression scale (CGI-S)[5] was used to assess the severity of burnout symptoms and changes from baseline. The scale is commonly used in clinical trials and allows clinicians to rate the severity of the patient's disease at the time of assessment through seven items scored from 1 ("*Normal, not at all ill*") to 7 ("*Among the most extremely ill patients*"). Finally, the Daily Ecological Momentary Assessment (EMA) is a smartphone tool to measure mood valence. The subjects have to respond every day (between 5 PM and 10 PM) to the App question "*On a 1-10 scale, how was your mood today?*" in a visual analog scale that ranges from 1 ("*lowest*") to 10 ("*highest*").[6]

© 2021 Crippa JAS et al. *JAMA Network Open*.

**eMethods 2.** Blood Tests, Quantification of Proinflammatory Cytokines, and Analysis of Plasma Levels of CBD

**Blood tests**

Full hemogram, sodium/potassium, urea, creatinine, total bilirubin, oxalacetic transaminase, pyruvic transaminase, gamma-glutamyltransferase, alkaline phosphatase, glucose, lipidogram, thyroid-stimulating hormone, testosterone, cortisol, estrogen, progesterone.

**Quantification of proinflammatory cytokines**

Proinflammatory cytokines, namely IL-1β and TNF-α levels, were measured with commercial ELISA kits DY201 and DY210 DuoSet, respectively (R&D Systems, Minneapolis, MN, USA) according to manufacturer's instructions. Briefly, human plasma samples were analyzed in 96-well microplates (DY990 - R&D Systems, Minneapolis, MN, USA) that were incubated overnight at room temperature with 100 µL of Capture antibodies (Mouse Anti-Human IL-1β or Mouse Anti-Human TNF-α) diluted in PBS (137 mM NaCl; 2.7 mM KCl; 8.1 mM Na2HPO4; 1.5 mM KH2PO4; pH 7.2-7.4, 0.2 µm filtered). After three washes with Wash Buffer (0.05% Tween® 20 in PBS, pH 7.2-7.4), microplates were blocked with 300 µL/well of Reagent Diluent (1% BSA in PBS, pH 7.2-7.4, 0.2 µm filtered) and incubated at room temperature for 1 hour. After another wash step, 100 µL of samples (previously centrifuged and diluted 1:10 in Reagent Diluent) or standards were added to the microplates and incubated for 4 hours at room temperature. Microplates were washed once again and 100 µL of Detection antibodies (Biotinylated Goat Anti-Human IL-1β or Biotinylated Goat Anti-Human TNF-α) diluted in Reagent Diluent were added and incubated for 2 hours at room temperature. After another wash step, 100 µL of Streptavidin conjugated to horseradish-peroxidase (Streptavidin-HRP) diluted in Reagent Diluent was added and incubated at room temperature, protected from light, for 20 minutes. The microplates were rewashed, and 100 µL/well of Substrate Solution (1:1 mixture of Color Reagent A: H2O2 and Color Reagent B: Tetramethylbenzidine) was added and then incubated for 20 minutes at room temperature. To stop the reaction, 50 µL of Stop Solution (H2SO4 2N) was added, and absorbance was immediately measured at 450 nm using a BioTek Epoch plate reader (BioTek Instruments, Winooski, VT, USA). Data analysis and calculations were performed with BioTek Gen5 3.05 and Microsoft Excel 2016 software.

**Analysis of plasma levels of CBD**

Plasma concentrations of CBD were determined using a Triple Quad API 5500™ liquid chromatography-mass spectrometry system (AB Sciex, Concord, ON, Canada) equipped with a high-performance liquid chromatography column Reversed-Phase UPLC® BEH C18 - 50x2.1mm, 1.7µm. The procedure has been validated to detect CBD concentrations ranging from 0.500 to 500.0 ng/mL, based on the analysis of 0.05 mL of plasma. The quantitation was performed using a weighted 1/x2 linear least-squares regression analysis generated from calibration standards. Quality control samples from high, medium, and low pools were processed with each sample run; the sample run was validated when at least two-thirds of the qualifying quality control samples were within 15% of their theoretical values and ≥ 50% of quality control samples at each level met this criterion.

© 2021 Crippa JAS et al. *JAMA Network Open*.

**eTable 1.** Score Differences Compared With Baseline

| Scale | Day | Mean difference | 95% Confidence | Partial |
|---|---|---|---|---|
| | | | Interval of the | Eta Square |
| | | | Difference | |
| | | | | |
| GAD-7 | 7 | 2.561 | 0.868 to 4.253 | O.072 |
| | 14 | 2.600 | 0.895 to 4.387 | 0.073 |
| | 21 | 2.611 | 0.750 to 4.472 | 0.63 |
| | 28 | 2.792 | 1.027 to 4.557 | 0.079 |
| | | | | |
| PHQ-9 | 7 | 1.999 | 0.454 to 3,545 | 0.054 |
| | 14 | 2.044 | 0.498 to 3.591 | 0.056 |
| | 21 | 2.548 | 0.953 to 4.143 | 0.080 |
| | 28 | 2,715 | 0.954 to 4.476 | 0.075 |
| | | | | |
| MBI | 7 | 0.519 | -2.190 to 3.228 | 0.001 |
| | 14 | 4.137 | 1.471 to6.803 | 0.076 |
| | 21 | 4.335 | 0.941 to 7.729 | 0.053 |
| | 28 | 4.009 | 0.430 to 7.589 | 0.041 |

© 2021 Crippa JAS et al. *JAMA Network Open*.

**eTable 2.** Laboratory Measurements

| Measurements | Cannabidiol | Standard Care |
|---|---|---|
| **Hematocrite (%)** | 14.2 (13.8 – 14.5) | 14.0 (13.6 – 14.3) |
| Baseline mean (95% C.I.) | 13.8 (13.5 – 14.2) | 13.8 (13.4 – 14.2) |
| Endpoint (95% C.I.) | (-2.1 – -.78) | (-1.45 – .16) |
| Endpoint – baseline (95% C.I.) | | |
| **Hemoglobin (g/cL)** | | |
| Baseline mean (95% C.I.) | 43.9 (43.0 – 44.9) | 43.4 (42.4 – 44.4) |
| Endpoint (95% C.I.) | 42.2 (41.1 - 43.3) | 42.8 (41.6 – 44.0) |
| Endpoint – baseline (95% C.I.) | (-.50 – -.10) | (-.38 – -.06) |
| **Total leucocytes (number/mm$^3$)** | | |
| Baseline mean (95% C.I.) | 6,165 (5,865 – 6,645) | 6,232 (5,743 – 6,721) |
| Endpoint (95% C.I.) | 10,468 (1,868 – 19,068) | 6,212 (5,794 – 6,630) |
| Endpoint – baseline (95% C.I.) | (-4,301 – 12,808) | (-476 – 464) |
| **Total lymphocytes (number/mm$^3$)** | 2,201 (2,034 – 2,368) | 2,305 (2,039 – 2,571) |
| Baseline mean (95% C.I.) | 2,132 (1,920 – 2,344) | 2,054 (1,888 – 2,221) |
| Endpoint (95% C.I.) | (-273 – 79.7) | (-499 – 4.9) |
| Endpoint – baseline (95% C.I.) | | |
| **Platelets (number/mm$^3$)** | | |
| Baseline mean (95% C.I.) | 277,412 (179,677 – 375,148) | 292,494 (200,065 – 384,922) |
| Endpoint (95% C.I.) | 238,573 (220,128 – 257,018) | 247,342 (228,131 – 266,554) |
| Endpoint – baseline (95% C.I.) | (-146,190 – 58,614) | (-138,342 – 46,164) |
| **Total cholesterol (mg/dL)** | | |
| Baseline mean (95% C.I.) | 177.2 (170-4 – 184.0) | 180.6 (172.2 – 189.0) |
| Endpoint (95% C.I.) | 180.5 (172.6 – 188.5) | 176.3 (167.6 – 185.1) |
| Endpoint – baseline (95% C.I.) | (-2.35 – 7.59) | (-9.8 0 – 2.47) |
| **HDL (mg/dL)** | | |
| Baseline mean (95% C.I.) | 50.7 (47.6 – 53.7) | 52.1 (48.3 – 55.8) |
| Endpoint (95% C.I.) | 108.4 (101-6 – 115.2) | 104.7 (98.1 – 111.3) |

© 2021 Crippa JAS et al. *JAMA Network Open*.

| | | |
|---|---|---|
| Endpoint – baseline (95% C.I.) | (-1.23 – 2.03) | (-3.5 – 7.59) |
| **LDL (mg/dL)** | | |
| Baseline mean (95% C.I.) | 105.9 (100.1 – 111.7) | 107.1 (100.0 – 114.2) |
| Endpoint (95% C.I.) | 108.4 (101.6 – 115.2) | 104.7 (98.1 – 111.3) |
| Endpoint – baseline (95% C.I.) | (-2.02 – 4.98) | (-6.8 – 3.04) |
| **VLDL (mg/dL)** | | |
| Baseline mean (95% C.I.) | 20.6 (18.1 – 23.2) | 21.4 (18.4 – 24.4) |
| Endpoint (95% C.I.) | 20.5 (18.5 – 22.6) | 23.2 (18.9 – 27.5) |
| Endpoint – baseline (95% C.I.) | (-1.67 – 1.91) | (-2.80 – 6.27) |
| **Triglycerides (mg/dL)** | | |
| Baseline mean (95% C.I.) | 109.0 (87.4 – 130.6) | 110.6 – 90.3 – 130.9) |
| Endpoint (95% C.I.) | 106.6 (90.0 – 123.3) | 113.9 (96.2 – 131.7) |
| Endpoint – baseline (95% C.I.) | (-16.28 – 14.44) | (-15.0 – 17.8) |
| **TGP (U/L)** | | |
| Baseline mean (95% C.I.) | 21.0 (18.6 – 23.4) | 21.6 (18.3 – 24.8) |
| Endpoint (95% C.I.) | 30.0 (18.0 – 42.0) | 24.3 (19.9 – 28.7) |
| Endpoint – baseline (95% C.I.) | (-3.00 – 21.92) | (.18 – 5.28) |
| **TGO (U/L)** | | |
| Baseline mean (95% C.I.) | 19.1 (17.9 – 20.2) | 19.2 (17.8 – 20.6) |
| Endpoint (95% C.I.) | 25.2 (17.1 – 33.3) | 20.4 (19.3 – 22.4) |
| Endpoint – baseline (95% C.I.) | (-2.1 – 14.6) | (-.2 – 2.7) |
| **Gamma-GT (UI/L)** | | |
| Baseline mean (95% C.I.) | 29.8 (21.0 – 38.6) | 29.1 (23.9 – 34.4) |
| Endpoint (95% C.I.) | 27.3 (22.2 – 32.3) | 31.8 (22.2 – 41.5) |
| Endpoint – baseline (95% C.I.) | (-2.86 – 5.82) | (-3.36 – 8.46) |
| **Alkaline phosphatase (U/L)** | | |
| Baseline mean (95% C.I.) | 62.6 (58.6 – 66.5) | 62.0 (57.2 – 66.9) |
| Endpoint (95% C.I.) | 67.2 (60.4 – 73.9) | 60.6 (55.8 – 65.4) |
| Endpoint – baseline (95% C.I.) | (-1.60 – 9.64) | (-3.48 – .311) |
| **Total bilirubin (mg/dL)** | | |
| Baseline mean (95% C.I.) | .63 (.54 – . 72) | .59 (.52 – .67) |
| Endpoint (95% C.I.) | .61 (.54 – .69) | .60 (.51 – .68) |

© 2021 Crippa JAS et al. *JAMA Network Open*.

| | | |
|---|---|---|
| Endpoint – baseline (95% C.I.) | (-.09 – -03) | (-.06 – .06) |
| **Sodium (mmol/L)** | | |
| Baseline mean (95% C.I.) | 139.9 (139.4 – 140.4) | 139.7 (139.2 – 140.1) |
| Endpoint (95% C.I.) | 136.6 (131.2 – 141.9) | 139.6 (139.0 – 140.1) |
| Endpoint – baseline (95% C.I.) | (-8.87 – 1.91) | (-.79 - .51) |
| **Potassium (mmol/L)** | | |
| Baseline mean (95% C.I.) | 4.3 (4.1 – 4.4) | 4.2 (4.1 – 4.3) |
| Endpoint (95% C.I.) | 4.2 (4.1 – 4.3) | 4.3 (4.1 – 4.4) |
| Endpoint – baseline (95% C.I.) | (-.32 – .11) | (-.14 – .16) |
| **Glycemia (mg/dL)** | | |
| Baseline mean (95% C.I.) | 90.5 (87.3 – 93.6) | 89.6 (86.6 – 92.5) |
| Endpoint (95% C.I.) | 92.5 (89.8 – 95.3) | 94.0 (91.3 – 96.7) |
| Endpoint – baseline (95% C.I.) | (-1.51 – 5.11) | (.91 – 7.91) |
| **TSH (UI/mL)** | | |
| Baseline mean (95% C.I.) | 1.7 (1.4 – 2.0) | 2.0 (1.7 – 2.3) |
| Endpoint (95% C.I.) | 1.9 (1.5 – 2.2) | 2.1 (1.8 – 2.5) |
| Endpoint – baseline (95% C.I.) | (-.10 - .47) | (-.10 - .38) |
| **Testosterone (ng/dL)** | | |
| Baseline mean (95% C.I.) | 404.4 (272.1 – 536.8) | 392.3 (279.0 – 505.7) |
| Endpoint (95% C.I.) | 451.6 (348.8 – 554.4) | 406.4 (229.0 – 513.8) |
| Endpoint – baseline (95% C.I.) | (-76.38 – 42.63) | (-97.24 – 62.06) |
| **Cortisol (ug/dL)** | | |
| Baseline mean (95% C.I.) | 11.4 (9.7 – 13.0) | 12.7 (11.0 – 14.4) |
| Endpoint (95% C.I.) | 10.4 (8.7 – 12.0) | 12.8 (10.8 – 14.9) |
| Endpoint – baseline (95% C.I.) | (-2.81 – .40) | (-1.80 – 1.92) |
| **Estradiol (pg/mL)** | | |
| Baseline mean (95% C.I.) | 96.3 (65.4 – 127.2) | 95.8 (58.5 – 133.0) |
| Endpoint (95% C.I.) | 103.8 (77.7 – 129.9) | 111.3 (50.2 – 172.4) |
| Endpoint – baseline (95% C.I.) | (-31.35 – 46.49) | (-32.70 – 17.89) |
| **Progesterone (ng/mL)** | | |
| Baseline mean (95% C.I.) | 2.4 (1.1 – 3.7) | 1.8 (.8 – 2.7) |
| Endpoint (95% C.I.) | 3.3 (1.3 – 5.2) | 2.2 (.9 – 3.4) |

© 2021 Crippa JAS et al. *JAMA Network Open*.

| | | |
|---|---|---|
| Endpoint – baseline (95% C.I.) | (-.90 – 2.06) | -.57 – 1.63 |
| **Creatinine (mg/dL)** | | |
| Baseline mean (95% C.I.) | .75 (.70 – .80) | .75 (.71 – .78) |
| Endpoint (95% C.I.) | .77 (.72 – .81) | .74 (.71 – . 78) |
| Endpoint – baseline (95% C.I.) | (-.01 – .05) | (-.02 – .02) |
| **Urea (mg/dL)** | | |
| Baseline mean (95% C.I.) | 32.1 (29.7 – 34.6) | 30.2 (27.9 – 32.6) |
| Endpoint (95% C.I.) | 31.6 (29.1 – 34.0) | 30.0 (27.7 – 32.4) |
| Endpoint – baseline (95% C.I.) | (-3.55 – .81) | (-2.18 – 1.84) |

© 2021 Crippa JAS et al. *JAMA Network Open*.

**eFigure 1.** Catchment Area of Ribeirão Preto, São Paulo State Region in the Health Regional Departments of São Paulo, Brazil

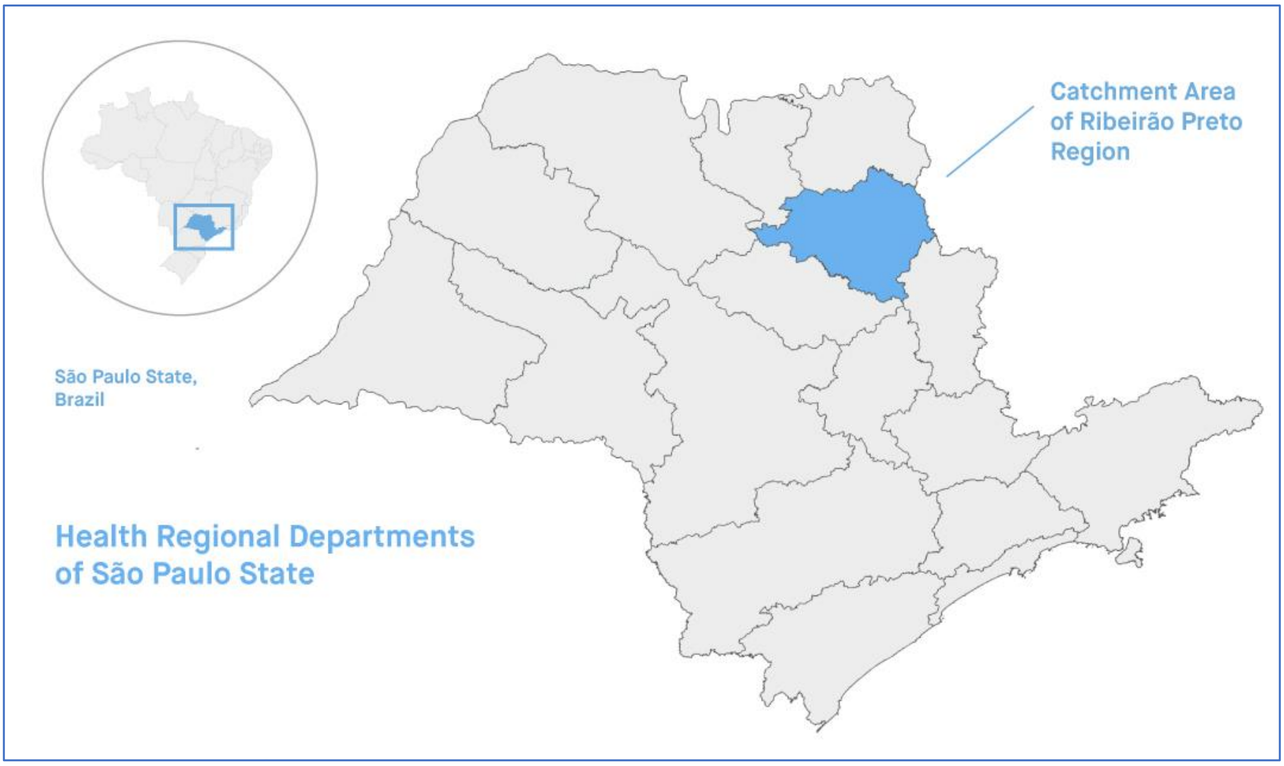


© 2021 Crippa JAS et al. *JAMA Network Open*.

**eFigure 2.** Cytokine Plasma Levels (IL-1β and TNF-α) Between Study Arms (Baseline vs Week 4)

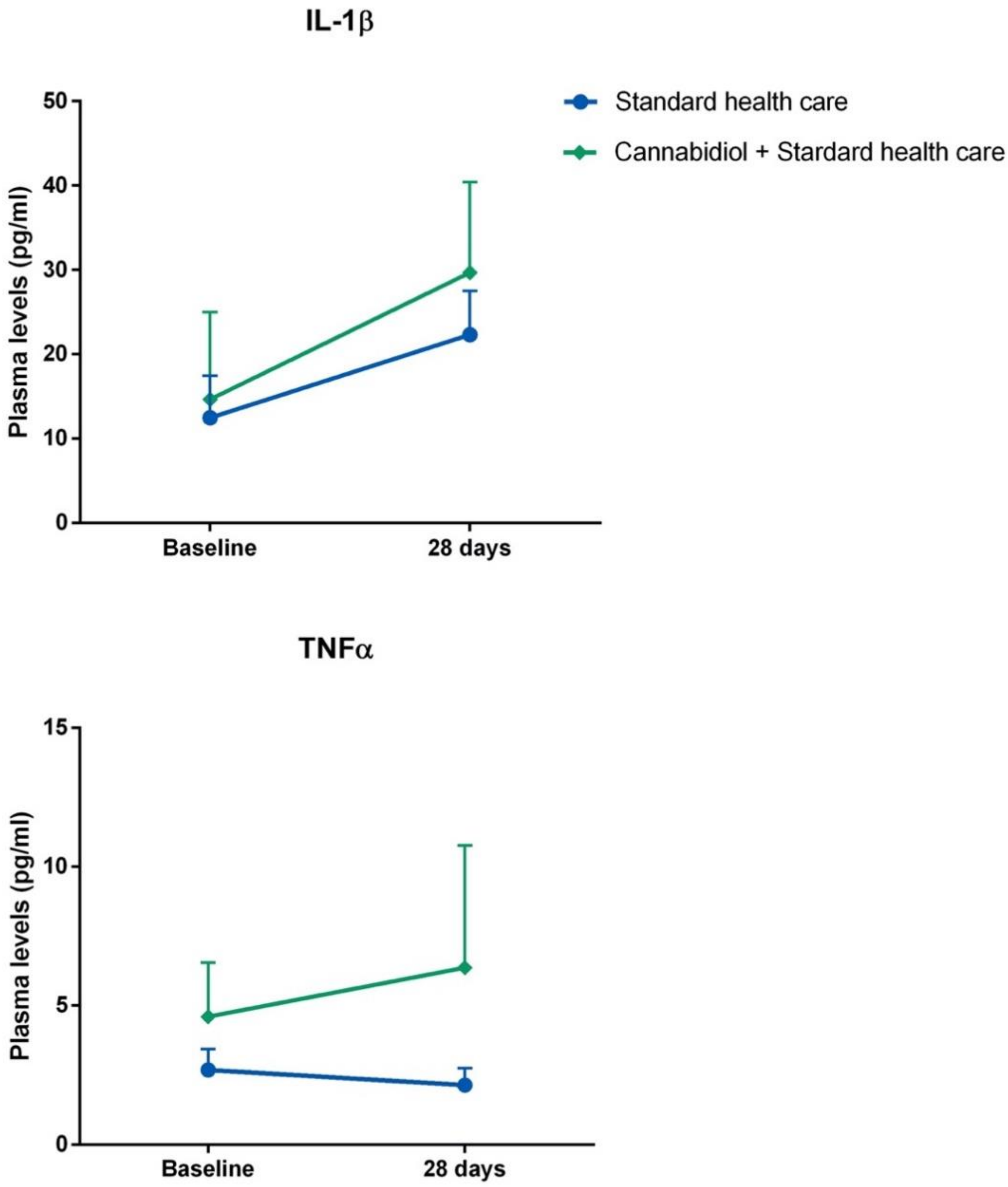


EDTA plasma levels of IL-1β and TNF-α of the subjects thar received standard health care (blue dots) or CBD + standard health care (green diamonds). Mean±SD. N=53/group.

© 2021 Crippa JAS et al. *JAMA Network Open*.

**eFigure 3.** CBD Plasma Levels at Time Points During Study

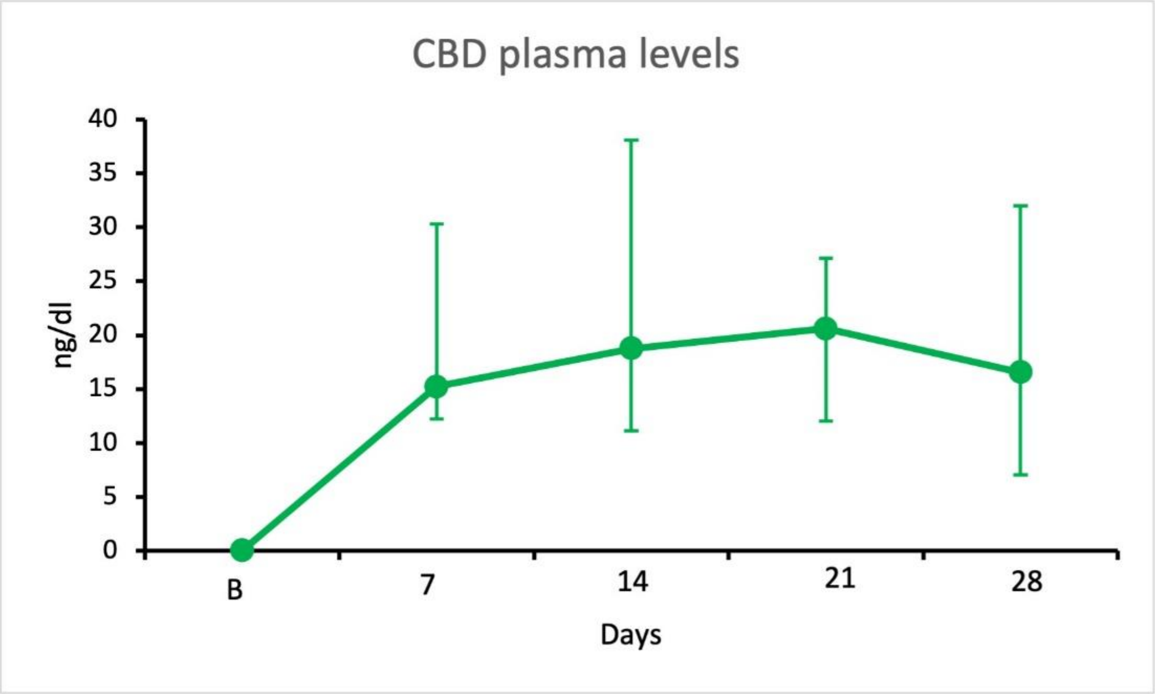


© 2021 Crippa JAS et al. *JAMA Network Open*.

**eFigure 4.** Most Common (>10%) Adverse Events in Study Arms

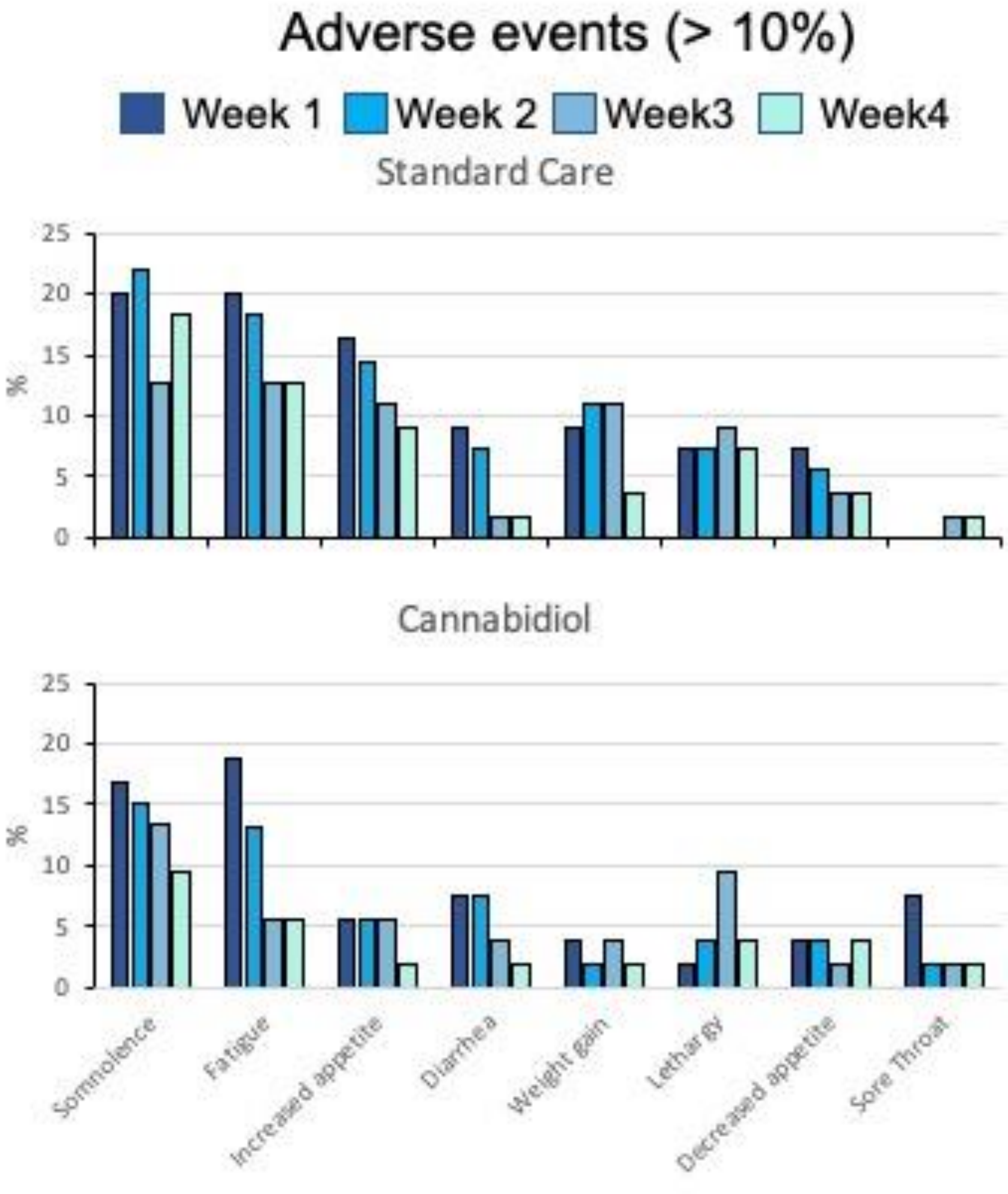


© 2021 Crippa JAS et al. *JAMA Network Open*.

**eReferences**


1. Carlotto MS, Câmara SG. Psychometrics properties of Maslach Burnout Inventory in a multifunctional sample. *Estud Psicol (Campinas)* 2007; 24(3): 325–32. doi: 10.1590/S0103-166X2007000300004
2. Moreno AL, DeSousa, DA, Souza AMFL, et al. Factor structure, reliability, and item parameters of the brazilian-portuguese version of the GAD-7 questionnaire. *Temas em Psicologia*, *24*(1), 367-376. doi: 10.9788/TP2016.1-25
3. Osório FL, Mendes AV, Crippa JA, Loureiro SR. Study of the discriminative validity of the PHQ-9 and PHQ-2 in a sample of Brazilian women in the context of primary health care. *Perspect Psychiatr Care*. 2009;45(3):216-227. doi:10.1111/j.1744-6163.2009.00224.x
4. Osório FL, Silva TDA, dos Santos RG, et al. Posttraumatic Stress Disorder Checklist for DSM-5 (PCL-5): transcultural adaptation of the Brazilian version. *Arch Clin Psychiatry* 2017; 44(1): 10–9. doi: 10.1590/0101-60830000000107
5. Lima MS, Soares BG, Paoliello G, et al. The Portuguese version of the Clinical Global Impression-Schizophrenia Scale: validation study. *Braz J Psychiatry*. 2007;29(3):246-249. doi:10.1590/s1516-44462007000300010
6. Foreman AC, Hall C, Bone K, Cheng J, Kaplin A. Just text me: using SMS technology for collaborative patient mood charting. J Participat Med. 2011 Sept 26; 3:e45.


© 2021 Crippa JAS et al. *JAMA Network Open*.
