# Supplementary material for: Efficacy and Safety of Cannabidiol Plus Standard Care vs Standard Care Alone for the Treatment of Emotional Exhaustion and Burnout Among Frontline Health Care Workers During the COVID-19 Pandemic: A Randomized Clinical Trial
Source: JAMA Netw Open. 2021 Aug 13;4(8):e2120603. doi: 10.1001/jamanetworkopen.2021.20603 (PMC8363917; doi:10.1001/jamanetworkopen.2021.20603)
Supplement: Supplement 3. — Nonauthor Collaborators [file jamanetwopen-e2120603-s003.pdf]

Supplemental Online Content: Nonauthor Collaborators

*Indicates required information. Only first name, last name, and suffix will appear in PubMed.

***Group Name(s):** Burnout and Distress Prevention With Cannabidiol in Front-line Health Care Workers Dealing With COVID-19 (BONSAI) Trial Investigators							
***First Name and Middle Initial(s)**	***Last Name**	***Suffix (eg, Jr, III)**	Academic Degrees	Institution	Location (city, state/province, country)	Role or Contribution, eg, chair, principal investigator	Group (if more than 1 Group listed in the byline) and/or Subgroup (eg, Steering Committee)
Debora C.	Litcanov	PD	Pharmacist	Faculdade de Ciências Farmacêuticas de Ribeirão Preto da Universidade de São Paulo	Ribeirão Preto, São Paulo/Brasil	Coordinator assistent	
Leonardo	Rodrigues	PD	Pharmacist	Faculdade de Ciências Farmacêuticas de Ribeirão Preto da Universidade de São Paulo	Ribeirão Preto, São Paulo/Brasil	Coordinator assistent	
Thiago F.	Alves	PD	Pharmacist	Faculdade de Ciências Farmacêuticas de Ribeirão Preto da Universidade de São Paulo	Ribeirão Preto, São Paulo/Brasil	Coordinator assistent	
Bárbara M.	Coutinho	PD	Pharmacist	Faculdade de Ciências Farmacêuticas de Ribeirão Preto da Universidade de São Paulo	Ribeirão Preto, São Paulo/Brasil	Pharmacist	
