# Supplementary material for: Efficacy and Safety of Cannabidiol Plus Standard Care vs Standard Care Alone for the Treatment of Emotional Exhaustion and Burnout Among Frontline Health Care Workers During the COVID-19 Pandemic: A Randomized Clinical Trial
Source: JAMA Netw Open. 2021 Aug 13;4(8):e2120603. doi: 10.1001/jamanetworkopen.2021.20603 (PMC8363917; doi:10.1001/jamanetworkopen.2021.20603)
Supplement: Supplement 4. — Data Sharing Statement [file jamanetwopen-e2120603-s004.pdf]

Data Sharing Statement

Crippa. Efficacy and Safety of Cannabidiol Plus Standard Care vs Standard Care Alone for the Treatment of Emotional Exhaustion and Burnout Among Frontline Health Care Workers During the COVID-19 Pandemic. *JAMA Netw Open*. Published August 13, 2021. doi:10.1001/jamanetworkopen.2021.20603

Data
Data available: Yes
Data types: Deidentified participant data
How to access data: Request for data must be sent to Prof Antonio Zuardi awzuardi@fmrp.usp.br
When available: With publication

Supporting Documents
Document types: Statistical/analytic code
How to access documents: Request for data must be sent to Prof Antonio Zuardi awzuardi@fmrp.usp.br
When available: With publication

Additional Information
Who can access the data: Request for data must be sent to Prof Antonio Zuardi awzuardi@fmrp.usp.br
Types of analyses: For metanalysis studies
Mechanisms of data availability: With investigator support, after approval of the proposal, and with a signed data access agreement. Moreover, the protocol must be accepted by both the local Ethical Committes
